# Endothelial protein C receptor-associated invasiveness of rheumatoid synovial fibroblasts is likely driven by group V secretory phospholipase A_2_

**DOI:** 10.1186/ar4473

**Published:** 2014-02-05

**Authors:** Meilang Xue, Kaitlin Shen, Kelly McKelvey, Juan Li, Yee-Ka Agnes Chan, Vicky Hatzis, Lyn March, Christopher B Little, Michael Tonkin, Christopher J Jackson

**Affiliations:** 1 Sutton Arthritis Research Laboratories, The University of Sydney at Royal North Shore Hospital, Pacific Highway, St Leonards, NSW 2065, Australia; 2 Department of Rheumatology, The University of Sydney at Royal North Shore Hospital, Pacific Highway, St Leonards, NSW 2065, Australia; 3 Raymond Purves Research Laboratory, The University of Sydney at Royal North Shore Hospital, Pacific Highway, St Leonards, NSW 2065, Australia; 4 Department of Surgery, Kolling Institute of Medical Research, The University of Sydney at Royal North Shore Hospital, Pacific Highway, St Leonards, NSW 2065, Australia

## Abstract

**Introduction:**

Rheumatoid synovial fibroblasts (RASFs) mediate joint inflammation and destruction in rheumatoid arthritis (RA). Endothelial protein C receptor (EPCR) is a specific receptor for the natural anticoagulant activated protein C (APC). It mediates the cytoprotective properties of APC and is expressed in rheumatoid synovial tissue. A recent report shows that group V secretory phospholipase A2 (sPLA_2_V) prevents APC from binding to EPCR in endothelium and inhibits EPCR/APC function. The aim of this study was to investigate the expression and function of EPCR on RASFs.

**Methods:**

Human synovial fibroblasts (SFs) were isolated from RA or osteoarthritis (OA) synovial tissues and treated with control, EPCR, or sPLA_2_V small interfering RNA (siRNA); recombinant human APC, tumor necrosis factor-alpha (TNF-α), or sPLA_2_V. RASF viability and migration/invasion were measured by 3-[4,5-dimethylthiazol-2-yl]-2,5-diphenyl tetrazolium bromide (MTT) and collagen gel migration/invasion assays, respectively, and cartilage degradation by 1,9-dimethylmethylene blue (DMMB) assay in the presence of human OA articular cartilage explants. The expression or activation of cytokines, EPCR, cadherin-11, mitogen-activated protein (MAP) kinases, and nuclear factor-kappa-B (NF-κB) or both were detected by enzyme-linked immunosorbent assay, Western blotting, or immunostaining.

**Results:**

EPCR was expressed by both OASFs and RASFs but was markedly increased in RASFs. When EPCR was suppressed by siRNA or blocking antibody cell viability, cell invasion and cartilage degradation were reduced by more than 30%. Inflammatory mediators interleukin-1-beta (IL-1β), cadherin-11, and NF-κB were significantly reduced by EPCR suppression under control or TNF-α-stimulated conditions. The expression or activation (or both) of MAP kinases ERK, p38, and JNK were also markedly decreased in cells transfected with EPCR siRNA. Further analysis revealed that sPLA_2_V co-localized with EPCR on RASFs. Suppression of sPLA_2_V reduced cell viability and cartilage degradation and increased APC binding to RASFs. Conversely, recombinant sPLA_2_V increased cartilage degradation, blocked APC binding to RASFs, and could not rescue the effects induced by EPCR suppression.

**Conclusions:**

Our results demonstrate that EPCR is overexpressed by RASFs and mediates the aggressive behavior of RASFs. This function of EPCR is contrary to its cytoprotective role in other settings and is likely driven by sPLA_2_V.

## Introduction

Rheumatoid arthritis (RA) is a chronic inflammatory disease characterized by synovial inflammation and hyperplasia, leading to progressive cartilage and bone destruction. Normal synovium forms a thin membrane at the edges of joints and provides lubrication and nutrients for the cartilage. In RA, this thin synovial lining layer dramatically increases and transforms into an inflammatory mass, known as the pannus [1,2]. This tissue mass expands and attaches to and invades the adjacent cartilage and subchondral bone, causing erosion. The major cell type accounting for the thickened lining layer and resultant pannus is the activated RA synovial fibroblasts (RASFs, also referred to as RA synoviocytes). As well as mediating tissue destruction, RASFs play a major role in catalyzing and sustaining RA by producing inflammatory cytokines such as interleukin-1-beta (IL-1β) and tumor necrosis factor-alpha (TNF-α), proangiogenic factors, and matrix-degrading enzymes [1,2]. Of equal concern, RASFs collaborate with and support the recruitment, survival, activation, and differentiation of T cells, B cells, macrophages, mast cells, osteoclasts, and endothelial cells throughout the RA synovium [1,2].

Once activated, the aggressive phenotype of RASFs can exist independent of inflammation. This was demonstrated by studies conducted in the severe combined immunodeficient mouse model of RA, in which implanted human RASFs degraded co-implanted human cartilage in the absence of inflammatory cells [3], and RASFs migrated via the bloodstream to implanted cartilage at a distant site, spreading RA to unaffected joints [4]. These data clearly indicates that RASFs are not passive bystanders, but are active participants in joint destruction in RA.

Endothelial protein C receptor (EPCR) is an endothelial transmembrane glycoprotein able to bind to a natural anticoagulant, protein C (PC), and its activated form, APC, with similar affinity [5]. Though originally identified as an endothelial cell receptor, EPCR has since been detected on many other cell types [6], including RA synovial lining cells [7]. As a receptor, EPCR mediates the majority of the anti-apoptotic, anti-inflammatory, and barrier-protective functions of APC [8]. In addition, EPCR itself is a central player in the convergent pathways of homeostasis and inflammation [8]. Recently, EPCR has been found to be overexpressed by some cancer cells and increased cancer cell migration and invasion [9-11]. However, the underlying mechanisms are not clear. EPCR can be cleaved from the cell surface to form soluble EPCR (sEPCR), which binds PC/APC with the same affinity as membrane-bound EPCR but blocks the protective function of APC [12-14]. Increased sEPCR is associated with many inflammatory/autoimmune diseases [15-17]. A recent report shows that group V secretory phospholipase A2 (sPLA_2_V) prevents APC binding to EPCR and inhibits EPCR/APC function by accommodating lysophosphatidylcholine (LysoPCh) or platelet-activating factor (PAF) in the hydrophobic groove of EPCR [18]. Being a key enzyme in the production of diverse mediators of inflammatory conditions, sPLA_2_V is present in significantly higher levels in the RA joint [19,20] and stimulates RA synovial proliferation and joint destruction [20,21].

The role of EPCR has not been studied in RASFs. We report here that EPCR is highly expressed by RASFs and contributes to the inflammatory and cartilage-degradative actions of these cells *in vitro*. Importantly, we show that these unexpected destructive effects of EPCR are likely driven by sPLA_2_V.

## Methods and materials

Fetal bovine serum (FBS), anti-cadherin 11 antibody, and TRIzol were purchased from Invitrogen (Carlsbad, CA, USA); anti-ERK, phosphorylated ERK (P-ERK), p38, P-p38 antibodies and anti-fibroblast marker (ER-TR7), A/G Plus-agarose, scrambled control, validated EPCR, PC, and sPLA_2_V small interfering RNAs (siRNAs) from Santa Cruz Biotechnology (Santa Cruz, CA, USA); recombinant APC from Eli Lilly and Company (Indianapolis, IN, USA); RiboCellin Transfection Reagents from BioCellChallenge (Paris, France); anti-β-actin and PC/APC antibodies and recombinant TNF-α from Sigma-Aldrich (St. Louis, MO, USA); anti-human CD68 from eBioscience (San Diego, CA, USA); human IL-1β, IL-6, and IL-8 enzyme-linked immunosorbent assay (ELISA) DuoSet from R&D Systems (Minneapolis, MN, USA); anti-nuclear factor-kappa B (anti-NF-κB) p65 subunit antibody from Chemicon International (Temecula, CA, USA); recombinant sPLA_2_V from Abcam (Cambridge, MA, USA); anti-human sPLA_2_V antibody from Cayman (Ann Arbor, MI, USA); NE-PER nuclear extraction reagents from Pierce Chemical Co. (Rockford, IL, USA). RCR252 (EPCR blocking antibody) and RCR92 (EPCR non-blocking antibody) were kindly provided by Kenji Fukudome (Saga Medical School, Saga, Japan).

### Synovial fibroblast isolation, culture, and treatment

Human SFs were isolated from synovial tissues obtained from knee replacement surgeries of seven patients with RA and eight patients with osteoarthritis (OA) (Table 1). After collagenase digestion, SFs were collected and cultured in Dulbecco’s modified Eagle’s medium (DMEM) supplemented with 10% FBS. Purity of RASFs was determined by flow cytometry stained with anti-CD68 antibody (eBioscience) and anti-fibroblast marker (ER-TR7). Cells were used for further experiments if more than 95% cells were positive for fibroblast marker. Passage 1 to 3 cells were used in all experiments. Confluent cells in 24-well plates were serum-starved with DMEM without FBS for 4 hours. After replacement with fresh DMEM, cells were treated with different reagents for 24 hours. Cells and culture supernatants were collected for further analysis. There was no difference in cell viability, detected by trypan blue exclusion and 3-[4,5-dimethylthiazol-2-yl]-2,5-diphenyl tetrazolium bromide (MTT) assays (see below for details), in response to different treatments for 24, 48, or 72 hours at the concentrations used for this study, compared with no treatment controls (data not shown).

**Table 1:** Demographics of patients from which synovial fibroblasts were isolated from synovial tissues obtained from knee replacement surgeries

	**Osteoarthritis**		**Rheumatoid arthritis**	
	**Female**	**Male**	**Female**	**Male**
Patients, number	3	5	5	2
Age, years	69.2 ± 7.2	71 ± 5.7	68.1 ± 6.2	70.5 ± 4.7

### Synovial fluid and cartilage

Synovial fluid samples were collected by needle puncture from the knee joints of 12 patients with RA and 12 patients with OA (Table 2). OA cartilage was obtained from knee/hip replacement surgeries.

**Table 2:** Demographics of patients from which synovial fluid samples were collected by needle puncture from the knee joints

	**Osteoarthritis**		**Rheumatoid arthritis**	
	**Female**	**Male**	**Female**	**Male**
Patients, number	9	3	5	7
Age, years	68.2 ± 5.1	70 ± 4.5	67.0 ± 4.6	69.5 ± 3.6

Usage of human tissue and fluid samples was in accordance with the ethics committee of the Northern Sydney Local Health District. All patients fulfilled the American College of Rheumatology criteria for RA and OA [22,23] and gave their written informed consent.

### siRNA transfection

SFs were transfected with EPCR, sPLA_2_V, or scrambled control siRNAs using RiboCellin Transfection Reagent in accordance with the protocol of the manufacturer. The efficacy of siRNA was detected by reverse-transcription real time polymerase chain reaction, ELISA, or Western blot.

### ELISA

EPCR, IL-1β, IL-6, and IL-8 in culture supernatants/whole cell lysates or synovial fluids were measured by ELISA DuoSet in accordance with the instructions of the manufacturer.

### Gelatin zymography

Matrix metalloproteinase-2 (MMP-2) and MMP-9 protein secretion and activation in the culture supernatants were measured by using gelatin zymography under non-reducing conditions [24].

### Immunoprecipitation and Western blot

SFs were lysed in cell lysis buffer (20 mM HEPES, 1% Triton X, and 10% Glycerol) supplemented with protease and phosphate inhibitors. Nuclear proteins were extracted with NE-PER nuclear extraction reagents in accordance with the instructions of the manufacturer. Immunoprecipitation (IP) was performed by using A/G Plus-agarose after incubation with anti-human EPCR antibody. Equal amounts of protein were separated on 10% or 15% (for IP) SDS-PAGE. Immunoreactivity was detected by using the ECL detection system and semi-quantified with gel image analysis software. Anti-human β-actin antibody was used to normalize equal loading.

### *In vitro* migration/invasion assay

RASF invasion was measured by using a collagen gel assay [25] with modifications. Briefly, RASFs (2 × 10^5^ cells/mL in DMEM) were mixed with equal volume of collagen solution (2 mg/mL, pH 7.2) and transferred into 24-well plates (20 μL/drop, 4 drops/well). After polymerization for 2 hours, 0.5 mL DMEM with 10% FBS was added to each well, and plates were incubated at 37°C. After 48 hours, cells and collagen gels were stained with crystal violet. Cells that had migrated out of gels were counted under a light microscope. At least 16 collagen gel drops per experimental group were analyzed.

### Immunohistochemistry, immunofluorescence, and toluidine blue staining

Cultured SFs in Falcon culture slides (Becton, Dickinson and Company, Franklin Lakes, NJ, USA) were fixed with 1% paraformaldehyde. Human synovial tissues were fixed in 10% phosphate-buffered saline-buffered formalin. Immunohistochemical and immunofluorescent staining and toluidine blue staining were performed as described previously [26,27]. Isotype IgG for each antibody was used as a negative control. Images were acquired and processed by using a digital camera and software (Nikon, Tokyo, Japan) and ImageJ.

### MTT assay

Cells (4 × 10^3^/well) were seeded into a 96-well plate. After 4 hours’ attachment, cells were treated with different test agents for 24, 48, or 72 hours, and cell viability was detected by the colorimetric MTT assay and confirmed by a trypan blue exclusion test.

### 1,9-dimethylmethylene blue assay

The level of sulphated glycosaminoglycans (sGAGs) released from the cartilage explants was determined by a 1,9-dimethylmethylene blue (DMMB) assay against a standard curve of chondroitin sulfate [28].

### Statistical analysis

Significance was determined by using one-way analysis of variance followed by Tukey’s honestly significant difference (HSD) *post hoc* test or Student *t* test. *P* values of less than 0.05 were considered statistically significant.

## Results

### EPCR is overexpressed by RASFs

**Figure 1:**
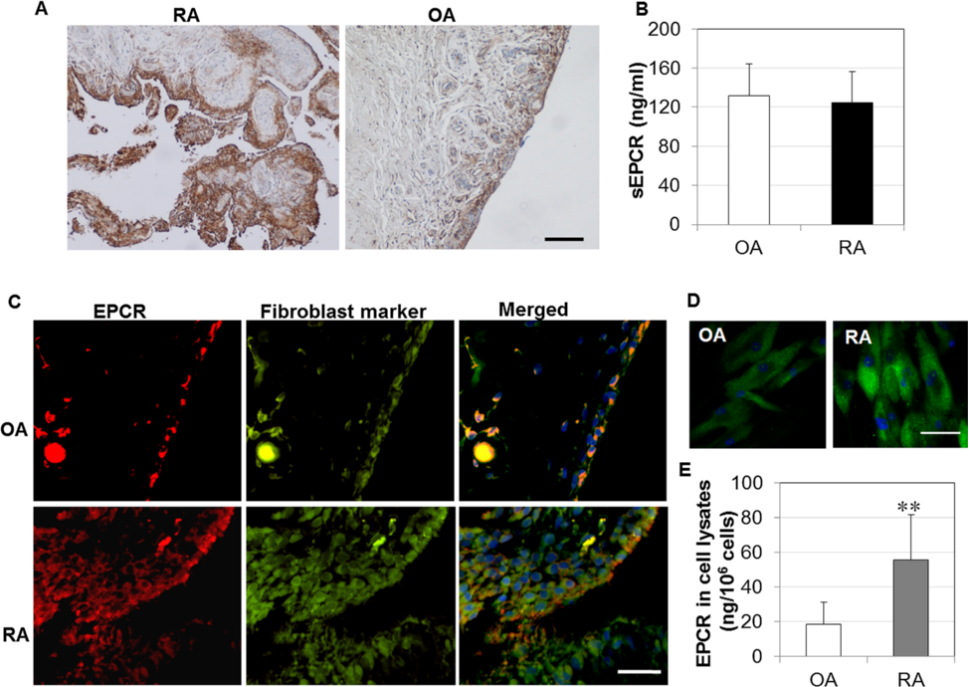
**Expression of endothelial protein C receptor (EPCR) in synovial tissues, synovial fluids, and cultured synovial fibroblasts (SFs). (A)** EPCR expression in synovial tissues from patients with rheumatoid arthritis (RA) and osteoarthritis (OA), detected by immunohistochemistry, and counterstained with Hematoxylin and Scott’s Bluing Solution. Scale bar: 400 μm. **(B)** Soluble EPCR (sEPCR) in synovial fluids from patients with OA and RA, detected by enzyme-linked immunosorbent assay (ELISA) and expressed as mean ± standard deviation (SD) (n = 12 each). **(C)** Co-localization of EPCR with the fibroblast marker, ER-TR7, in synovial tissues from patients with RA and OA, detected by immunofluorescent staining. Scale bar: 50 μm. **(D)** EPCR expression in cultured SFs, detected by immunofluorescent staining. Scale bar: 20 μm. Nuclei were counterstained with 4′-6-diamidino-2-phenylindole (DAPI) (blue) in (C) and (D). The images represent one of three experiments. **(E)** EPCR in whole cell lysates of cultured SFs detected by ELISA and expressed as mean ± SD (n = 7 each). Data was analyzed by Student *t* test, **P <0.01.

ECPR expression by synovial tissues was determined by immunostaining. There was a stronger staining in RA synovial tissue than in OA tissue (Figure 1A). Most EPCR staining was localized to the lining and sub-lining layers. As expected, blood vessels were positively stained for EPCR. Interestingly, there was no difference in the levels of sEPCR, which could bind PC/APC and inhibit the function of APC [14], in synovial fluids from RA versus OA patients (Figure 1B).

To confirm whether SFs express EPCR, dual immunofluorescent staining was performed by using anti-EPCR antibody and the fibroblast marker, ER-TR7. EPCR staining in the lining layer was localized on SFs in both OA and RA synovium (Figure 1C). Isolated RASFs also expressed higher levels of EPCR than OASFs as assessed by immunostaining (Figure 1D). ELISA data using whole cell lysates confirmed that RASFs expressed threefold higher EPCR than OASFs (Figure 1E). sEPCR was not detectable in culture supernatants of RASFs or OASFs. At the gene level, both OASFs and RASFs expressed EPCR mRNA, with RASFs expressing more than 50% higher levels than OASFs at passage 1.

### Suppressing EPCR inhibits the aggressive properties of RASFs

**Figure 2:**
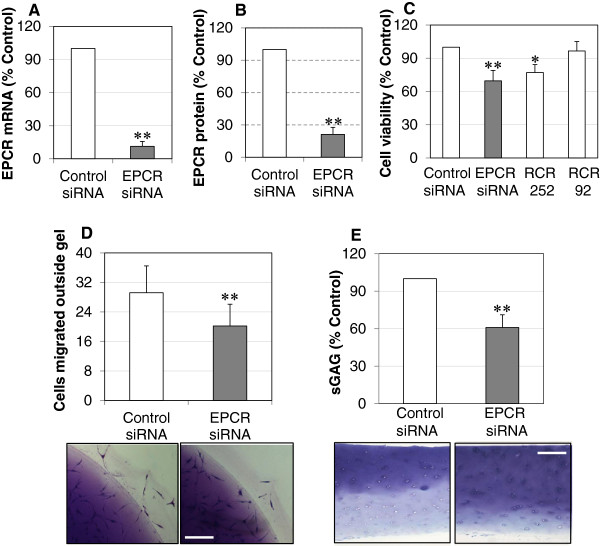
**Blocking endothelial protein C receptor (EPCR) suppresses rheumatoid synovial fibroblast (RASF) survival/growth, migration/invasion, and the ability to degrade cartilage. (A)** EPCR mRNA levels in RASFs transfected with EPCR or control small interfering RNA (siRNA) for 24 hours and detected by reverse-transcription real time polymerase chain reaction (PCR). **(B)** EPCR protein in whole cell lysates of RASFs transfected with EPCR or control siRNA for 72 hours and detected by enzyme-linked immunosorbent assay (ELISA). **(C)** Viability of RASFs transfected with control or EPCR siRNA or EPCR blocking (RCR252) or non-blocking (RCR92) antibody for 72 hours and detected by colorimetric 3-[4,5-dimethylthiazol-2-yl]-2,5-diphenyl tetrazolium bromide (MTT) and trypan blue exclusion assays. **(D)** Migration/invasion of RASFs transfected with control or EPCR siRNA for 72 hours. After siRNA transfection for 24 hours, RASFs were embedded into collagen gels at 1 × 10^5^ cells/mL. After incubation for another 48 hours, collagen gels were stained and cells that had migrated outside of gels were counted (16 gel drops for each treatment). **(E)** Cartilage degradation by RASFs transfected with control or EPCR siRNA. After 24 hours of siRNA transfection, OA cartilage was co-incubated with RASFs for another 24 hours, and culture medium was then collected for measuring sulphated glycosaminoglycans (sGAGs) by a 1,9-dimethylmethylene blue (DMMB) assay. Cartilage explants were fixed and processed for toluidine blue staining. Data on each graph are shown as mean ± standard deviation (SD) from four RASF cell lines derived from four RA synovial tissues and analyzed by one-way analysis of variance followed by Tukey’s honestly significant difference (HSD) *post hoc* test (C) or Student *t* test (A,B,D,E), compared with control siRNA. The images represent one of three experiments Scale bar: 100 μm. *P <0.05, **P <0.01.

To examine whether EPCR is associated with the aggressive properties of RASFs, EPCR expression was suppressed by its specific siRNA or function was blocked by the blocking antibody RCR252. The efficacy of siRNA was confirmed by reverse transcription-polymerase chain reaction and ELISA, which suppressed gene expression by more than 85% at 24 hours and protein levels by more than 75% at 72 hours after transfection (Figure 2A,B).

RASF survival/growth, a critical step that leads to synovial hyperplasia, was measured at 72 hours after transfection. Suppressing EPCR with siRNA resulted in a more than 30% reduction in RASF viability (Figure 2C). This was confirmed by the blocking antibody RCR252 treatment, which displayed a significant reduction in viable cells, whereas the non-blocking control antibody, RCR92, had no effect (Figure 2C). Another aggressive property of RASFs is their elevated migratory/invasive capacity. To assess this, a collagen gel migration/invasion assay was performed. In basal conditions, 20% to 30% of cells migrated out of a gel after 48 hours of incubation. There were more than 30% fewer cells migrating out of the collagen gel following EPCR siRNA transfection compared with control siRNA (Figure 2D), indicating that suppressing EPCR inhibits RASF migration/invasion. EPCR siRNA transfection also reduced sGAG release, a measure of cartilage degradation [29], into culture media from co-incubated cartilage explants, by more than 40% (Figure 2E). Toluidine blue staining confirmed that cartilage explants preserved more cartilage when incubated with EPCR siRNA-transfected RASFs when compared with control siRNA (Figure 2E).

### Suppressing EPCR reduces MMP-2, IL-1β, and cadherin-11 and inactivates NF-κB and MAP kinases

**Figure 3:**
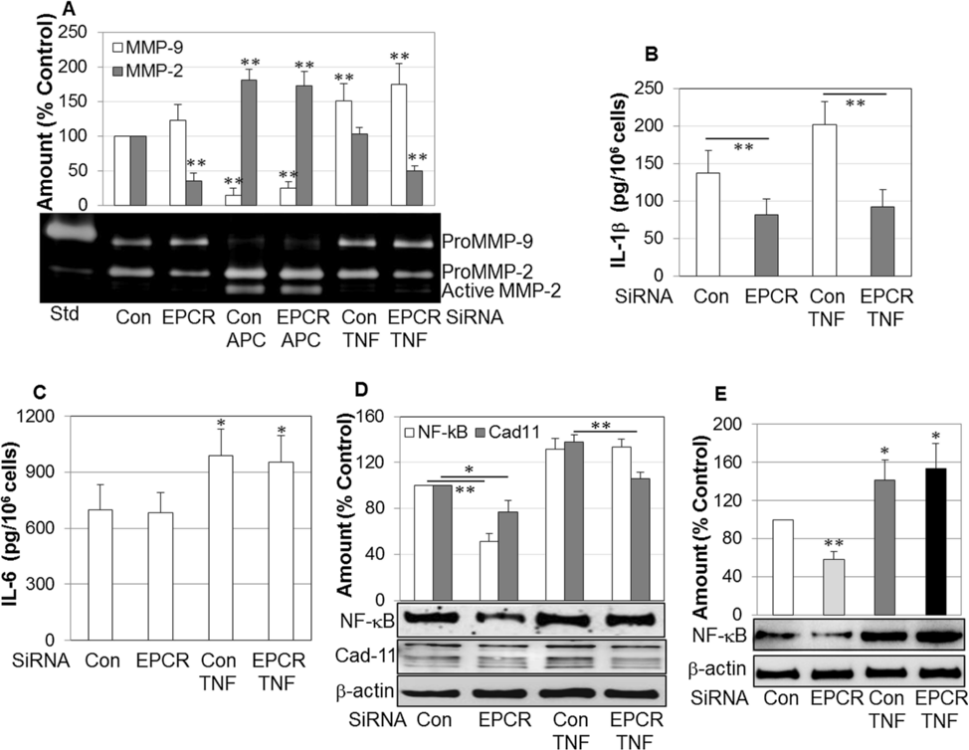
**Effect of endothelial protein C receptor (EPCR) blockage on the expression/activation of matrix metalloproteinases (MMPs), interleukin-1-beta (IL-1β), IL-6, and nuclear factor-kappa-B (NF-κB) by rheumatoid synovial fibroblasts (RASFs).** RASFs were transfected with control (Con) or EPCR small interfering RNA (siRNA) for 24 hours. Medium was then replaced with Dulbecco’s modified Eagle’s medium (DMEM) without fetal bovine serum (FBS) and treated with tumor necrosis factor-alpha (TNF-α) (100 ng/mL) or activated protein C (APC) (10 μg/mL) for a further 24 hours. Media were collected for gelatin zymography to detect MMP-2 and MMP-9, and total MMP-2 and MMP-9 were semi-quantified by image analysis software in comparison with control **(A)** or enzyme-linked immunosorbent assay (ELISA) to quantify IL-1β **(B)** and IL-6 **(C)**. Cells were collected and lysed for Western blot analysis of NF-κB or cadherin-11 (or both) in whole cell lysates **(D)** and in nuclei **(E)** and semi-quantified by image analysis software in comparison with β-actin. Std, MMP-2 and MMP-9 standard. Data on graph are shown as mean ± standard deviation (SD) of three different RASF cell lines and analyzed by using analysis of variance followed by Tukey’s honestly significant difference (HSD) *post hoc* test. *P <0.05, **P <0.01, compared with relevant control.

The invasive properties of RASFs are associated with their ability to produce higher levels of inflammatory cytokines and matrix-degrading enzymes and excessive activation of inflammatory signaling molecules such as NF-κB [1,2]. Inhibition of EPCR had no significant effect on matrix-degrading enzyme MMP-9; however, MMP-2 was decreased by 60% when detected by gelatin zymography (Figure 3A). TNF-α had a minimal effect on MMP-2 but stimulated MMP-9 in both control and EPCR siRNA-transfected cells (Figure 3A). In contrast, APC nearly completely inhibited MMP-9 but stimulated and activated MMP-2. Inhibition of EPCR did not affect the ability of APC to activate MMP-2 (Figure 3A), which is consistent with the ability of APC to directly activate MMP-2 [30].

In response to EPCR siRNA transfection, secretion of IL-1β, the key initiator of inflammation and cartilage breakdown in RA [31,32], was reduced by approximately 50% from RASFs (Figure 3B). TNF-α stimulated IL-1β production in control but not in EPCR siRNA-transfected RASFs (Figure 3B). This inhibitory effect was cytokine-specific for IL-1β as silencing EPCR by siRNA had no effect on IL-6 in either control or TNF-α-stimulated conditions (Figure 3C).

Silencing EPCR also reduced the activation of NF-κB in whole cell lysates by 50% in control but not after TNF-α stimulation (Figure 3D). This was confirmed by the activation of NF-κB in the nuclear fraction, which displayed a reduction similar to that shown in whole cell lysate (Figure 3E). In addition, cadherin-11, a key molecule that regulates RASF function [33], was significantly suppressed when RASFs were transfected with EPCR siRNA in both control and TNF-α-stimulated conditions (Figure 3D).

**Figure 4:**
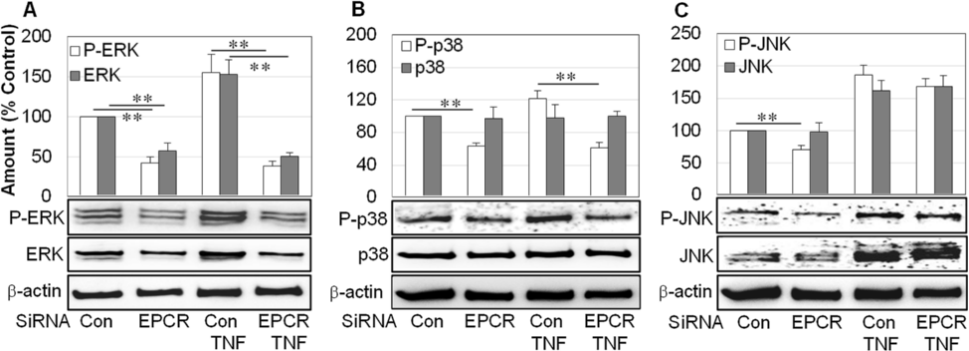
**Effect of endothelial protein C receptor (EPCR) blockage on the expression/activation of mitogen-activated protein (MAP) kinase ERK, p38, and JNK in rheumatoid synovial fibroblasts (RASFs).** RASFs were transfected with control (Con) or EPCR small interfering RNA (siRNA) for 24 hours. Medium was then replaced with Dulbecco’s modified Eagle’s medium (DMEM) without fetal bovine serum (FBS) and treated with tumor necrosis factor-alpha (TNF-α) (100 ng/mL) for a further 24 hours. Cells were collected and lysed for Western blot analysis and semi-quantified by image analysis software to measure ERK and P-ERK **(A)**, p38 and P-p38 **(B)**, and JNK and P-JNK **(C)**; β-actin was included as an internal standard. The gel images represent one of three experiments. Data on graphs are shown as mean ± standard deviation (SD) of three different RASF cell lines and were analyzed by analysis of variance followed by Tukey’s honestly significant difference (HSD) *post hoc* test. **P <0.01. Relative comparisons are indicated by horizontal lines.

The expression and activation of mitogen-activated protein (MAP) kinases ERK, p38, and JNK are important in the regulation of RASF survival/growth and inflammation [34-38]. Silencing EPCR inhibited total expression and activation of ERK by more than 50% (Figure 4A). Although TNF-α stimulated and activated ERK under otherwise basal conditions, it had no effect on ERK when cells were transfected with EPCR siRNA. EPCR siRNA transfection selectively inhibited p38 activation, but not the non-activated form, in the presence or absence of TNF-α (Figure 4B). Silencing EPCR suppressed the activation of JNK in basal conditions but not after TNF-α stimulation (Figure 4C).

### sPLA_2_V co-localizes with EPCR in synovial tissues and blocks APC binding

**Figure 5:**
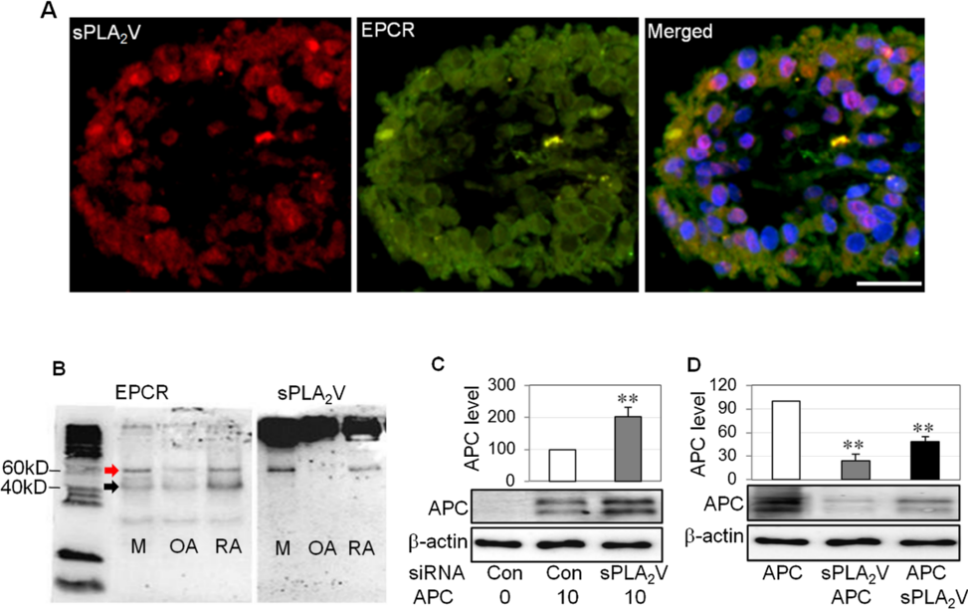
**Group V secretory phospholipase A**_**2 **_**(sPLA**_**2**_**V) is co-localized with endothelial protein C receptor (EPCR) and blocks activated protein C (APC) binding to rheumatoid synovial fibroblasts (RASFs). (A)** sPLA_2_V was co-localized with EPCR in rheumatoid arthritis (RA) synovial tissue, detected by immunofluorescent staining. Nuclei were counterstained by 4′-6-diamidino-2-phenylindole (DAPI) (blue). Scale bar: 50 μm. **(B)** sPLA_2_V and EPCR expression by MCF-7 cells (M, used as a positive control), RASFs (RA), or OASFs (OA), detected by immunoprecipitation using anti-EPCR antibody and followed by Western blot using anti-EPCR or sPLA_2_V antibody under non-reducing conditions. Red arrow indicates EPCR and sPLA_2_V complex. Black arrow indicates EPCR. **(C)** APC in whole cell lysates of RASFs transfected with small interfering RNA (siRNA) for control (Con) or sPLA_2_V siRNA for 48 hours and treated with APC (10 μg/mL) for 24 hours and detected by Western blot. **(D)** APC in the whole cell lysates of RASFs treated with recombinant APC (2 μg/mL), recombinant sPLA_2_V (2 μg/mL) for first 30 minutes then APC (sPLA_2_V + APC) or APC for first 30 minutes then sPLA_2_V (APC + sPLA_2_V) for 4 hours, detected by Western blot. The images represent one of three different experiments using three different RASF cell lines. Data on (C) and (D) were semi-quantified by image analysis software in comparison with β-actin, expressed as a percentage of control and shown as mean ± standard deviation (SD) (n = 4) and analyzed by one-way analysis of variance followed by Tukey’s honestly significant difference (HSD) *post hoc* test. **P <0.01.

The above findings suggest that EPCR promotes inflammation in RA, which is contrary to its well-described anti-inflammatory effects [8]. A recent study showed that sPLA_2_V inhibits EPCR’s cytoprotective function in endothelial cells by preventing APC binding to EPCR [18]. We explored whether SPLA_2_V was involved in EPCR’s inflammatory actions on RASFs. Dual immunofluorescent staining suggested that SPLA_2_V was co-localized with EPCR in synovial tissues (Figure 5A). In culture, co-immunoprecipitation of cell lysates with anti-EPCR antibody followed by Western blotting to detect EPCR generated a band corresponding to EPCR (black arrow) and another band at approximately 60 kD which was the complex of EPCR and sPLA_2_V (red arrow) (Figure 5B). Further detection of the same membrane with anti-sPLA_2_V antibody confirmed that the upper band was the EPCR and sPLA_2_V complex (Figure 5B). These results indicate that EPCR and sPLA_2_V can bind together on RASFs. To investigate whether sPLA_2_V could prevent the binding of APC to RASFs, we used two approaches. First, endogenous sPLA_2_V was suppressed by siRNA for 48 hours, and APC (10 μg/mL) was added to cells for 4 hours. Western blot analysis showed that membrane-associated APC was increased in cells transfected with sPLA_2_V siRNA when compared with cells transfected with control siRNA (Figure 5C). Second, when RASFs were pre-incubated with recombinant sPLA_2_V before the addition of APC, there was markedly less cell-associated APC compared with APC alone or with addition of APC prior to sPLA_2_V (Figure 5D). These data suggest that sPLA_2_V prevents APC binding to RASFs.

### sPLA_2_V promotes the aggressive properties of RASFs via EPCR

**Figure 6:**
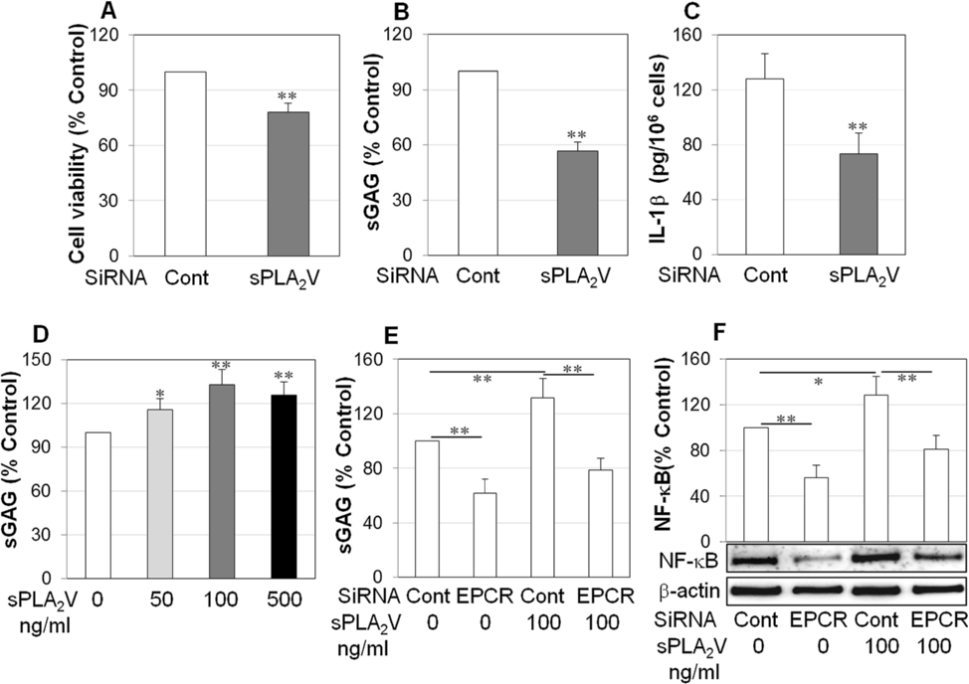
**Group V secretory phospholipase A**_**2 **_**(sPLA**_**2**_**V) promotes the aggressive properties of rheumatoid synovial fibroblasts (RASFs) via endothelial protein C receptor (EPCR).** RASFs were transfected with control or sPLA_2_V small interfering RNA (siRNA). **(A)** RASF viability after siRNA transfection for 72 hours, detected by colorimetric 3-[4,5-dimethylthiazol-2-yl]-2,5-diphenyl tetrazolium bromide (MTT) assay and confirmed by trypan blue exclusion assay. **(B)** Cartilage degradation by RASFs. After siRNA transfection for 24 hours, medium was replaced with Dulbecco’s modified Eagle’s medium (DMEM) without fetal bovine serum (FBS), and osteoarthritis (OA) cartilage explants were incubated with RASFs for a further 24 hours. Media were collected for detection of sulphated glycosaminoglycans (sGAGs) by 1,9-dimethylmethylene blue (DMMB) assay. **(C)** Interleukin-1-beta (IL-1β) protein in the culture media of RASFs transfected with control or sPLA_2_V siRNA. After siRNA transfection for 24 hours, medium was replaced with DMEM without FBS, RASFs were incubated for a further 24 hours, and media were collected for enzyme-linked immunosorbent assay (ELISA). **(D)** Cartilage degradation ability of RASFs in response to recombinant sPLA_2_V (50, 100, and 500 ng/mL) for 24 hours, measured by DMMB assay. **(E)** Cartilage-degradative ability of RASFs transfected with EPCR siRNA for 48 hours and recombinant sPLA_2_V (100 ng/mL) for 24 hours, measured by DMMB assay. **(F)** NF-κB activation in whole cell lysates of RASFs transfected with EPCR siRNA for 48 hours and recombinant sPLA_2_V (100 ng/mL) for 24 hours and semi-quantified by image analysis software in comparison with β-actin. The images represent one of three experiments. Data on graph are shown as mean ± standard deviation (SD) of three or four different RASF cell lines. Data were analyzed by Student *t* test (A-C) by one-way analysis of variance (D-F), followed by Tukey’s HSD *post hoc* test. *P <0.05, **P <0.01 when compared with relevant control.

To examine whether sPLA_2_V regulates the aggressive properties of RASFs, cell viability and cartilage degradation were examined after transfection with sPLA_2_V siRNA. Silencing sPLA_2_V significantly reduced cell viability by more than 20% (Figure 6A), cartilage degradation by more than 40% (Figure 6B), and IL-1β production by more than 40% (Figure 6C). Furthermore, recombinant sPLA_2_V at 100 ng/mL promoted RASF-mediated cartilage degradation by approximately 30% (Figure 6D) but did not significantly raise RASF viability after 72 hours of treatment (data not shown). To investigate whether sPLA_2_V-associated aggressive properties of RASFs are mediated by EPCR, RASFs were transfected with EPCR siRNA and stimulated with recombinant sPLA_2_V. sPLA_2_V significantly increased RASF-mediated sGAG release and NF-κB activation in control cells (Figure 6E and F); however, there was a 40% reduction in sPLA_2_V-stimulated sGAG release and 45% reduction in sPLA_2_V-stimulated NF-κB activation in EPCR siRNA-transfected cells (Figure 6E and F).

Taken together, these data suggest that sPLA_2_V is mainly responsible for RASF-mediated cartilage degradation and inflammation associated with overexpression of EPCR.

## Discussion

SFs are key effectors in the pathogenesis of RA. In this study, we have demonstrated that RASFs express higher levels of EPCR than OASFs. In contrast to its conventional anti-inflammatory effects, EPCR expressed by RASFs was associated with increased invasiveness and inflammatory responses of these cells. Further investigation revealed that sPLA_2_V is co-localized with EPCR, prevents APC from interacting with EPCR, and drives EPCR-associated invasiveness, inflammation, and cartilage degradation.

EPCR plays a critical role in augmenting PC activation and mediating the anti-inflammatory and cytoprotective functions of the PC pathway [39]. In this study, however, EPCR expression was associated with the destructive effects of RASFs. Suppressing EPCR decreased RASF viability, invasion, and cartilage degradation ability through inhibition of inflammatory cytokine IL-1β. In RA, IL-1β stimulates the production of MMPs and the maturation of osteoclasts and ultimately promotes cartilage breakdown [31,32] and the development of bone erosion [40,41]. In synovium, IL-1β is a major activator of SFs by promoting the activation of NF-κB and MAP kinases [1]. Moreover, IL-1β, but not TNF-α, can markedly induce sPLA_2_V production by SFs [21].

Suppression of EPCR also markedly reduced the expression and activation of MAP kinases which regulate cell survival, apoptosis, viability, cellular stress, and inflammatory responses. The three major classes of MAP kinases—ERK, p38, and JNK—are all enhanced in RA synovial tissues [37,38]. Survival/growth of RASFs is mediated by ERK [35], which plays an important role in the maintenance of RA by promoting pannus formation [34]. JNK activation is required for the regulation of collagenase production by SFs [35,36]. ERK and JNK activation predict development of erosive disease in early arthritis [35,36]. Our results suggest that EPCR promotes RASF viability and inflammation via activation of MAP kinases. Inhibition of NF-κB and cadherin-11 by suppression of EPCR also likely contributes to reduced invasion and cartilage degradation by RASFs [33,42]. Our data also show that suppressing EPCR in RASFs inhibits production of MMP-2, a function of EPCR that is present in endothelial cells [43]. Although MMP-2 may promote cartilage degradation, it suppresses the development of inflammatory joint disease in a mouse arthritis model [44]. MMP-2 is constitutively expressed by RASFs, and the exact role of MMP-2 activity by these cells is still unclear.

Our unexpected finding that EPCR is not cytoprotective in RASFs has precedent in cancer cells. EPCR increases cell migration and invasion of breast cancer cells *in vitro*[9] and is a possible biomarker of ovarian cancer onset [10]. EPCR also promotes metastasis and correlates with clinical outcome in lung adenocarcinoma [11]. However, vascular wall EPCR inhibits cancer cell adhesion and transmigration [45]. The reasons for these contradictory findings are unclear but may reflect the different regulatory mechanisms of EPCR in different cell types and tissues.

EPCR can be regulated by proteolytic release from the cell surface to form sEPCR [12,13]. sEPCR binds PC to inhibit APC generation or binds APC to block the protective function of APC [14]. Pro-inflammatory cytokines IL-1β and TNF-α enhance EPCR shedding from the endothelial cell surface [16,46]. Accordingly, higher levels of sEPCR have been reported in patients with systemic inflammatory diseases [15,17,47]. sEPCR produced by ovarian cancer cells is a possible biomarker of cancer onset [10] and is likely to be a biomarker of cancer-associated hypercoagulability in human hematologic malignancies [48]. However, in the present study, we found that cell-associated EPCR is three times higher in cultured RASFs than in OASFs and that there is no difference in sEPCR, either in cultured supernatants of OASFs and RASFs or in synovial fluids from patients with OA and RA (Figure 1). These data suggest that it is not sEPCR, but the membrane bound form, that exerts the inflammatory and cartilage-degradative actions of RASFs. This destructive property of EPCR differs from its cytoprotective actions in other settings [49]. The present study explored the reasons for this diametrical role of EPCR in RA. Although RASFs express PC/APC, neither silencing endogenous PC by siRNA nor adding recombinant APC significantly changed RASF viability (Additional file 1 shows this in more detail), indicating that this function of EPCR is not due to PC/APC. We found that this paradox may be explained by the actions of sPLA_2_V, which generates bioactive lipids LysoPCh and PAF. These two lipids can substitute for PCh, which normally resides in the deep groove of EPCR and is required for normal EPCR function. When LysoPCh or PAF substitutes for PCh, they impair the ability of EPCR to interact with PC or APC, thus inhibiting EPCR’s cytoprotective function in endothelial cells [18].

In the present study, RASFs expressed high levels of sPLA_2_V, which promoted the aggressive properties of RASFs. Suppressing endogenous sPLA_2_V reduced RASF viability, cartilage degradation ability, and IL-1β, whereas recombinant sPLA_2_V enhanced RASF-mediated cartilage degradation and NF-κB activation (Figure 6). Further study using dual immunostaining and co-immunoprecipitation indicated that sPLA_2_V and EPCR are spatially associated with each other on RASFs. We found that sPLA_2_V not only blocks APC binding to RASFs but also uses EPCR to promote its inflammatory effects on RASFs. This was evidenced by the fact that suppressing endogenous sPLA_2_V enhanced but that recombinant sPLA_2_V inhibited APC binding to RASFs (Figure 5). Moreover, suppression of EPCR reduced the stimulatory effect of sPLA_2_V on cartilage degradation and NF-κB activation by RASFs (Figure 6). We propose that, similar to its effect on endothelial cells [18], sPLA2V impairs the ability of EPCR to interact with APC in RASFs, inhibiting EPCR’s cytoprotective function.

## Conclusions

In summary, this study demonstrated that elevated EPCR promotes the inflammatory responses and invasiveness of RASFs, which are likely driven by sPLA_2_V. These results provide new insights into the mechanisms underlying SF-mediated joint inflammation in RA and may inspire new targeted therapeutic approaches.

## Abbreviations

APC: activated protein C; DMEM: Dulbecco’s modified Eagle’s medium; ELISA: enzyme-linked immunosorbent assay; EPCR: endothelial protein C receptor; FBS: fetal bovine serum; IL: interleukin; IP: immunoprecipitation; LysoPCh: lysophosphotidylcholine; MAP: mitogen-activated protein; MMP: matrix metalloproteinase; MTT: colorimetric 3-[4,5-dimethylthiazol-2-yl]-2,5-diphenyl tetrazolium bromide; NF-κB: nuclear factor-kappa-B; OA: osteoarthritis; PAF: platelet-activating factor; PC: protein C; RA: rheumatoid arthritis; RASF: rheumatoid synovial fibroblast; sEPCR: soluble endothelial protein C receptor; SF: synovial fibroblast; sGAG: sulphated glycosaminoglycan; siRNA: small interfering RNA; sPLA2V: group V secretory phospholipase A_2_; TNF-α: tumor necrosis factor-alpha.

## Competing interests

The authors declare that they have no competing interests.

## Authors’ contributions

MX participated in conception and design, data acquisition, collection and analysis, manuscript writing, and final approval of the manuscript. KS, KM, JL, Y-KAC, and LM participated in data acquisition, collection and analysis, and critical revision and final approval of the manuscript. VH, CBL, and MT participated in data acquisition, analysis, and critical revision and final approval of the manuscript. CJJ participated in data acquisition, collection and analysis, manuscript writing, and critical revision and final approval of the manuscript. All authors read and approved the final manuscript.

## Supplementary Material

Additional file 1: Figure S1 Protein C/activated protein C (PC/APC) expression and its effect on rheumatoid synovial fibroblast (RASF) viability. Click here for file
