# Supplementary material for: Endothelial protein C receptor-associated invasiveness of rheumatoid synovial fibroblasts is likely driven by group V secretory phospholipase A2
Source: Arthritis Res Ther. 2014 Feb 5;16(1):R44. doi: 10.1186/ar4473 (PMC3979138; doi:10.1186/ar4473)
Supplement: Additional file 1: Figure S1 — Protein C/activated protein C (PC/APC) expression and its effect on rheumatoid synovial fibroblast (RASF) viability. [file ar4473-S1.pptx]

## Slide 1
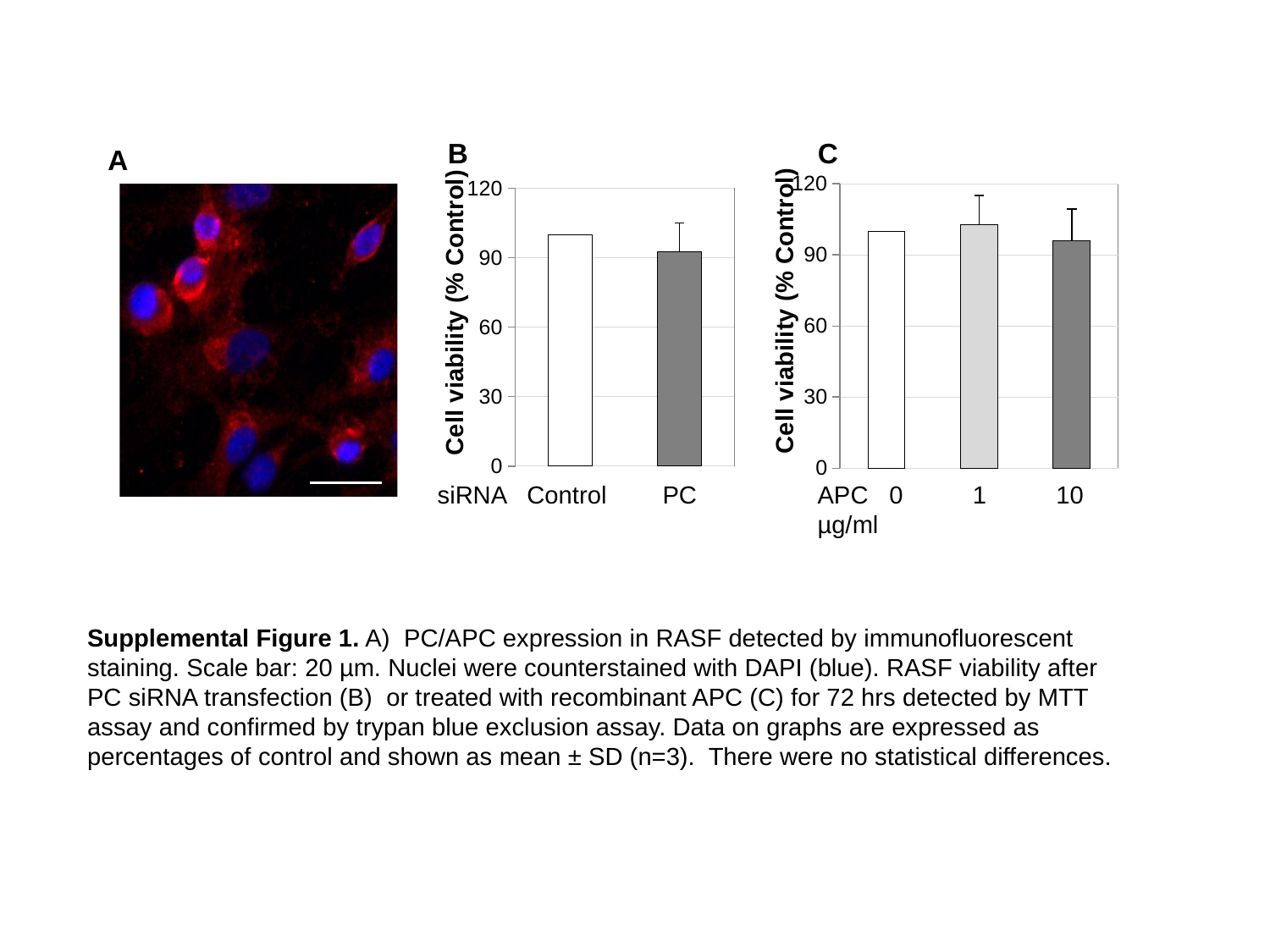

B
C
A
### Chart
| Category | |
|---|---|
| Control | 100.0 |
| A1 | 102.56611243072 |
| A10 | 95.9224069675376 |
### Chart
| Category | |
|---|---|
| ConsiRNA | 100.0 |
| PCsiRNA | 92.4562 |
Cell viability (% Control)
Cell viability (% Control)
siRNA Control PC
APC 0 1 10 µg/ml
Supplemental Figure 1. A) PC/APC expression in RASF detected by immunofluorescent staining. Scale bar: 20 µm. Nuclei were counterstained with DAPI (blue). RASF viability after PC siRNA transfection (B) or treated with recombinant APC (C) for 72 hrs detected by MTT assay and confirmed by trypan blue exclusion assay. Data on graphs are expressed as percentages of control and shown as mean ± SD (n=3). There were no statistical differences.
